# Resting-state fMRI seizure onset localization meta-analysis: comparing rs-fMRI to other modalities including surgical outcomes

**DOI:** 10.3389/fnimg.2024.1481858

**Published:** 2024-12-17

**Authors:** Varina L. Boerwinkle, Mary A. Nowlen, Jesus E. Vazquez, Martin A. Arhin, William R. Reuther, Emilio G. Cediel, Patrick J. McCarty, Iliana Manjón, Jubran H. Jubran, Ashley C. Guest, Kirsten D. Gillette, Frances M. Nowlen, Andrew R Pines, Meitra H. Kazemi, Bahjat F. Qaqish

**Affiliations:** ^1^Division of Child Neurology, University of North Carolina, School of Medicine, Chapel Hill, NC, United States; ^2^Department of Obstetrics and Gynecology, Banner University Medical Center, Phoenix, AZ, United States; ^3^Department of Biostatistics, University of North Carolina at Chapel Hill, Chapel Hill, NC, United States; ^4^University of North Carolina, School of Medicine, Chapel Hill, NC, United States; ^5^Surgical Neurology Branch, National Institute of Neurological Disorders and Stroke, Bethesda, MD, United States; ^6^Tulane University, School of Medicine, New Orleans, LA, United States; ^7^Department of Psychiatry, University of Arizona College of Medicine, Phoenix, AZ, United States; ^8^Department of Neurosurgery, University of California, San Diego, San Diego, CA, United States; ^9^University of Arizona College of Medicine, Phoenix, AZ, United States; ^10^Creighton University School of Medicine, Phoenix, AZ, United States; ^11^Department of Psychiatry, Brigham & Women’s Hospital, Boston, MA, United States

**Keywords:** meta-analysis, resting-state functional MRI, seizure network, epilepsy surgery, neuroimaging

## Abstract

**Objective:**

Resting-state functional MRI (rs-fMRI) may localize the seizure onset zone (SOZ) for epilepsy surgery, when compared to intracranial EEG and surgical outcomes, per a prior meta-analysis. Our goals were to further characterize this agreement, by broadening the queried rs-fMRI analysis subtypes, comparative modalities, and same-modality comparisons, hypothesizing SOZ-signal strength may overcome this heterogeneity.

**Methods:**

PubMed, Embase, Scopus, Web of Science, and Google Scholar between April 2010 and April 2020 via PRISMA guidelines for SOZ-to-established-modalities were screened. Odd ratios measured agreement between SOZ and other modalities. Fixed- and random-effects analyses evaluated heterogeneity of odd ratios, with the former evaluating differences in agreement across modalities and same-modality studies.

**Results:**

In total, 9,550 of 14,384 were non-duplicative articles and 25 met inclusion criteria. Comparative modalities were EEG 7, surgical outcome 6, intracranial EEG 5, anatomical MRI 4, EEG-fMRI 2, and magnetoencephalography 1. Independent component analysis 9 and seed-based analysis 8 were top rs-fMRI methods. Study-level odds ratio heterogeneity in both the fixed- and random-effects analysis was significant (*p* < 0.001). Marked cross-modality and same-modality systematic differences in agreement between rs-fMRI and the comparator were present (*p* = 0.005 and *p* = 0.002), respectively, with surgical outcomes having higher agreement than EEG (*p* = 0.002) and iEEG (*p* = 0.007). The estimated population mean sensitivity and specificity were 0.91 and 0.09, with predicted values across studies ranging from 0.44 to 0.96 and 0.02 to 0.67, respectively.

**Significance:**

We evaluated centrality and heterogeneity in SOZ agreement between rs-fMRI and comparative modalities using a wider variety of rs-fMRI analyzing subtypes and comparative modalities, compared to prior. Strong evidence for between-study differences in the agreement odds ratio was shown by both the fixed- and the random-effects analyses, attributed to rs-fMRI analysis variability. Agreement with rs-fMRI differed by modality type, with surgical outcomes having higher agreement than EEG and iEEG. Overall, sensitivity was high, but specificity was low, which may be attributed in part to differences between other modalities.

## Highlights

- Heterogeneity in study-level rs-fMRI SOZ analysis methods and comparative SOZ-localizing modalities likely contributed to lack of study-level agreement odds ratios.
- Agreement in SOZ localization with rs-fMRI varied between modalities and within studies using the same modality, suggesting heterogeneity in agreement.
- The level of agreement in SOZ localization was higher for surgical outcome when compared to EEG and iEEG.
- Operationalizing resting-state fMRI (rs-fMRI) for epilepsy surgery is hindered by variability in analysis methods and validation benchmarks. Currently, only independent component analysis and accepted gold standards are validated at the meta-analysis level for seizure onset localization.

## 1 Introduction

Epilepsy is a serious neurologic disorder affecting over 70 million people with a lifetime prevalence of 7.6 per 1,000 persons worldwide that is characterized by recurrent and spontaneous seizures (Fiest et al., 2014; Thijs et al., 2019). Epilepsy is associated with social stigma, multiple comorbidities, and a high economic burden (Fiest et al., 2014). Nearly 80% of people with this disorder live in low- and middle-income countries (Beghi, 2020), and many remain untreated (Fiest et al., 2014).

In 30% of drug-resistant epilepsy (DRE) cases, medications fail to achieve seizure control (Kalilani et al., 2018). Thus, surgical resection of the seizure onset zone (SOZ) is a potentially curative option, improving their quality of life (Kalilani et al., 2018; Engel et al., 2003; Jobst and Cascino, 2015). However, accurate localization of SOZ, the brain region of seizure origin, is vital for successful surgical outcomes (Fiest et al., 2014). Surgical intervention fails in 30–70%, depending on SOZ-localizing modalities used and epilepsy subtype (Bulacio et al., 2012; Engel, 2016; Gonzalez-Martinez et al., 2007; Laxer et al., 2014; Malmgren and Edelvik, 2017; McIntosh et al., 2004; Sillanpaa and Shinnar, 2010; Covidence systematic review software, n.d.). Despite the curative or palliative effects, surgery is an underutilized resource, potentially due to lack of or late provider referral, negative views on the likelihood of seizure freedom, and fears of associated risks (Thijs et al., 2019; Galan et al., 2021).

The non-invasive SOZ modalities include scalp electroencephalography (EEG), magnetoencephalography (MEG), positron emission tomography (PET), ictal single-photon emission computed tomography (SPECT), high-resolution magnetic resonance imaging (MRI), and resting-state functional MRI (rs-fMRI) (Thijs et al., 2019; Sheng et al., 2018). Contrastingly, invasive means of SOZ localization use electrodes on the surface or inserted into brain tissue, termed intracranial EEG (iEEG) or stereo encephalography (SEEG).

EEG is useful for localizing the SOZ based on the pattern and location of electrical signals; however, the most common form, scalp EEG, is limited by relatively poor spatial resolution and is needed to capture ictal activity. Simultaneous EEG-fMRI can provide improved spatial resolution compared to scalp EEG but also requires specialized equipment. Ictal SPECT studies utilize tracers to identify the SOZ as regions of hyper-perfusion and may be combined with interictal images and overlayed with MRI (Perissinotti et al., 2018). Similarly, combined acquisition of PET/MRI has been shown to improve identification of epileptogenic foci compared to PET alone (Boscolo Galazzo et al., 2016; Khalaf et al., 2022). One major limitation of both EEG and SPECT is that they require a patient to be actively seizing to effectively localize the SOZ. MEG detects the magnetic fields generated by neuronal electrical currents but is primarily limited in its ability to detect deeper brain structures frequently associated with DRE (Vivekananda et al., 2021). aMRI can identify structural abnormalities leading to epileptic activity but does not delineate the epileptic activity.

When non-invasive investigations fail or need verification to accurately identify the SOZ, invasive diagnostic modalities such as iEEG are often necessary. iEEG involves a subdural grid or strip electrodes to capture activity from relatively large surfaces of the brain or depth electrodes that can reach the deep brain structures (Burneo et al., 2006; Grande et al., 2020; Jayakar et al., 2016; Nagahama et al., 2018). iEEG is the current gold standard for presurgical SOZ localization; however, its invasive nature results in higher mortality and morbidity than non-invasive methods (Chakraborty et al., 2020). While morbidity rates have decreased over time, patients would benefit from a more reliable non-invasive method that could reduce the regions requiring iEEG confirmation or increase the likelihood of placing SEEG in the true SOZ.

Comparatively, rs-fMRI is both non-invasive and can be performed during the interictal period. Rs-fMRI is an emerging modality that can be used to detect the SOZ and map the surrounding eloquent brain areas. Due to its non-invasiveness and potential for whole brain network detection, rs-fMRI has delineated pathophysiology of various neurologic and psychiatric disorders (Cha et al., 2015; Gong et al., 2020; Ibrahim et al., 2021; Lau et al., 2019; Pini et al., 2020). Rs-fMRI assesses functional connectivity through the detection of variation of blood-oxygen-level-dependent (BOLD) signals that reflect differences in neural activity (Matthews and Jezzard, 2004).

There are various methods for assessing rs-fMRI, and it is unknown which are most useful for SOZ localization. For example, independent component analysis (ICA), which is a data-driven method, yields the whole brain network profile. Contrastingly, there are hypothesis-driven methods, such as seed-based correlation (SBC) maps, which evaluate only those regions pre-determined to be of interest (Metwali and Samii, 2019; Rosazza et al., 2014; Rosazza and Minati, 2011). Other commonly used methods for localizing activity include regional homogeneity (ReHo) and amplitude of low-frequency fluctuation (ALFF)/fractional ALFF (fALFF) (Zang et al., 2015). ReHo measures the synchronization of neighboring voxels, and ALFF/fALFF measures the amplitude of time series fluctuations from regions of interest (Zang et al., 2015; Zang et al., 2004; Zou et al., 2008). Further advantages and limitations in general of the various rs-fMRI signal processing methods are reported (Cole et al., 2010).

Armed with such methods, there has been an emergence of rs-fMRI in clinical settings (Anzellotti et al., 2010; Chen et al., 2017; Khoo et al., 2019; Reyes et al., 2016; Zhao et al., 2019). However, there are few large or meta-studies validating rs-fMRI findings in epilepsy compared to controls or other pathology (Zang et al., 2015). Furthermore, there is only one meta-analysis evaluating the localization of seizure networks for the purpose of epilepsy surgery evaluation by Chakraborty et al. (2020). Their study compared the SOZ localization by rs-fMRI with ICA and primary component analysis (PCA) to that by intracranial EEG or epilepsy surgery outcomes, finding non-inferiority of rs-fMRI. While positive agreement in SOZ localization between rs-fMRI and the comparative modalities was found, the study also found moderate heterogeneity in agreement between studies.

Thus, the first goal of this study is to evaluate the validity of rs-fMRI in localizing the SOZ, by comparing it to other established SOZ-localizing modalities across a diverse set of analysis approaches and validation standards. The second goal is to evaluate the clinical relevance of rs-fMRI for pre-surgical planning by evaluating its agreement with surgical outcomes. With these two goals, this study aims to clarify the role of rs-fMRI in epilepsy surgery and provide further insights into its potential as a valid, non-invasive modality for SOZ localization. We hypothesized that the underlying BOLD–epilepsy neuronal relationship may exhibit a sufficiently large signal to overcome the heterogeneity introduced by both the choice of rs-fMRI analysis method and the physiological properties of the validating SOZ modalities.

## 2 Methods

### 2.1 Search strategy

A literature review was conducted using the Preferred Reporting Items for Systematic Reviews and Meta-Analyses (PRISMA) guidelines. PubMed, Embase, Scopus, Web of Science, and the first 100 results of Google Scholar were evaluated up to 22 April 2020. The search was limited to publications after 2000. Terms queried consisted of variations of resting-state fMRI, epilepsy, seizure, irritative zone, network mapping, lateral, local, and connectivity network, resulting in *n* = 14,382. Two additional resources were added by hand searching. The articles were loaded into Covidence (Veritas Health Innovation),^1^ an online software designed to streamline literature reviews for meta-analyses. After duplicates were removed, 9,550 articles remained. The abstracts and full-text articles were each screened by two of ten independent reviewers, and discrepancies were reviewed by one of two independent reviewers blinded to the original votes as a tiebreaker. Within Covidence, each article was then randomly assigned to the independent interviewers.

### 2.2 Eligibility criteria

Study inclusion criteria consisted of (1) primary research, (2) involving human patients with DRE, (3) patients who underwent rs-fMRI, and (4) the rs-fMRI results were compared to another SOZ-localizing modality. Exclusion criteria were non-peer-reviewed publication abstracts with *n* < 10 (if published then any number of subjects were allowed), studies written in a language other than English, unavailable subject level SOZ data, duplicate populations with another included article (with exception as detailed in the results that also have novel population), or studies that did not focus on localizing the SOZ.

### 2.3 Data extraction

Each study was analyzed for SOZ localization by a prior non-rs-fMRI established modality, SOZ localization by rs-fMRI, and surgical outcomes, if applicable. The comparative modalities include anatomical MRI, EEG, EEG-fMRI, MEG, iEEG, and surgical outcomes. When a study used multiple comparative modalities, only one primary modality was selected for analysis. The selected modality had to meet the following criteria: (1) It provided usable results for all study participants, and (2) it was described by the authors as the primary modality that influenced the surgical target location. In cases where no primary modality was specified, the comparative modalities were chosen in the following category order: (1) surgical outcomes, (2) iEEG, (3) EEG-fMRI, EEG, MEG, or anatomical MRI. In the case of the third category, the modality with the most detailed subject level data and the most specific brain location information was selected. Applying this process returned the following distribution of primary comparative modalities: iEEG/EEG (193 cases, 43%), surgical outcomes (154 cases, 34%), and anatomical MRI (80 cases, 18%).

True positives (TP) were defined as the SOZ, and rs-fMRI and comparative modality localized to the same place. False positives (FP) were defined as when the rs-fMRI localized a SOZ where a comparative modality did not detect a SOZ. False negatives (FN) were defined as when the comparative modality localized to a different location OR the comparative modality localized a SOZ while rs-fMRI did not detect any. True negatives (TN) were defined as when both rs-fMRI and the comparative modality did not localize a SOZ. For each study, the counts of the TP, FP, FN, and TN were extracted and tabulated into 2×2 tables, with rs-fMRI as the rows and the comparative as the columns (Table 1).

**Table 1:** Patient description of defined rs-fMRI SOZ truth for patients without surgical outcomes.

		Comparative modality localized potential SOZ	
		Positive	Negative
rs-fMRI Localized Potential SOZ	Positive	True positives Both rs-fMRI and the comparative modality localized the SOZ to the same region	False positives rs-fMRI localized an SOZ where the comparative modality did not detect any
	Negative	False negatives Comparative modality localized to a different region or localized an SOZ while rs-fMRI did not detect any	True negatives Neither modality localized an SOZ

This table definitions were used for studies that compared rs-fMRI to intracranial EEG, EEG-fMRI, EEG, MEG, or anatomical MRI.

If surgical outcomes were available, which is considered the highest level of proof of validity of SOZ, then SOZ TP, FP, TN, and FN were defined according to Boerwinkle et al. (2017) as summarized in Table 2.

**Table 2:** Patient description of defined rs-fMRI SOZ truth for patients with surgical outcomes.

		Surgical outcome	
		Positive	Negative
rs-fMRI SOZ Prediction	Positive	True positives rs-fMRI SOZ resected, outcome Engel 1 or rs-fMRI SOZ resected, and agreed with iEEG SOZ, outcome Engel 2–3* or rs-fMRI SOZ anatomically seperate from iEEG, and rs-fMRI SOZ not resected, outcome Engel 3–4**	False positives All rs-fMRI SOZs resected, agreed with iEEG, but outcome <50% seizure reduction and Engel 3,4 or rs-fMRI SOZ not resected, did not agree with iEEG, outcome Engel 1–3
	Negative	False negatives rs-fMRI SOZ not detected but iEEG detected SOZ, outcome Engel 1–3	True negatives Neither rs-fMRI SOZ nor iEEG SOZ detected, outcome Engel 3–4

*Meaning moderately good seizure outcome, thus region resected, which did align with both the rs-fMRI and iEEG SOZ was involved with the seizure generation/propagation significantly enough to impact surgical outcome. **Meaning the outcome was poor, so the region targeted by the surgery was likely not significantly involved in the seizure generation/propagation. And since the rs-fMRI SOZ was NOT resected, then there is a potential that it could still be a significant seizure generation/propagation region. This table definitions were used for studies that compared rs-fMRI to surgical outcomes as denoted by Engel Classifications.

### 2.4 Assessment of bias

The Quality Assessment of Diagnostic Studies (QUADAS-2) checklist was used to assess the quality of included articles (Whiting et al., 2011). A 0 denotes low risk, a 1 denotes high risk, and a 2 denotes an uncertain risk. These findings are displayed in Table 3, with final expert review denoted in the caption.

**Table 3:** QUADAS ratings of studies included in the meta-analysis.

	Domain 1: Patient selection		Domain 2: Index test		Domain 3: Reference standard		Domain 4: flow and timing
	Could the selection of patients have introduced bias?	Is there concern that the included patients do not match the review question?	Could the conduct or interpretation of the resting state have introduced bias?	Is there concern that the index test, its conduct, or interpretation differ from the review question?	Could the reference standard, its conduct, or its interpretation have introduced bias?	Is there concern that the target condition as defined by the reference standard does not match the review question?	Could the patient flow have introduced bias?
Anzellotti et al. (2010)	1*	0	0	0	0	0	0
Barron et al. (2015)	0	0	0	0	0	0	0
Bettus et al. (2010)	0	0	0	0	0	0	0
Boerwinkle et al. (2017)	0	0	0	0	0	0	0
Boerwinkle et al. (2019)	0	0	0	0	0	0	0
Chen et al. (2017)	0	0	0	0	0	0	0
Gnanadas et al. (2017)	2	0	2	0	2	0	2
Hunyadi et al. (2014)	0	0	0	0	0	0	0
Hunyadi et al. (2015a)	0	0	0	0	0	0	0
Hunyadi et al. (2015b)	0	0	0	0	0	0	0
Jann et al. (2008)	0	0	2	0	0	0	0
Kang et al. (2003)	1*	0	0	0	0	0	0
Khoo et al. (2019)	0	1^**t**^	0	0	2	0	0
Lee et al. (2014)	0	0	0	0	0	0	0
Morgan et al. (2004)	1*	0	0	0	0	0	0
Reyes et al. (2016)	0	0	2	0	2	0	0
Wang et al. (2007)	1*	0	0	0	0	0	0
Song et al. (2006)	1*	0	0	0	0	0	0
Stufflebeam et al. (2011)	1*	0	0	0	0	0	0
Su et al. (2015)	0	0	0	0	0	0	0
Tavares et al. (2017)	1*	0	0	0	0	0	0
van Houdt et al. (2015)	0	0	0	0	0	0	0
Weaver et al. (2013)	0	0	0	0	0	0	0
Yang et al. (2015)	0	0	0	0	0	0	0
Zhao et al. (2019)	0	0	0	0	1**	0	2

0 = low risk (green); 1 = high risk (pink); 2 = uncertain risk (yellow); *Selection of patients that was not well specified as consecutive or reasoning otherwise were rated as a 1. ** Six patients who underwent SEEG and rs-fMRI were analyzed by predefined metrics. Thus, it is unclear why our interpreters rated this study as possibly biased, since it removed expert opinion from interpretation. And in retrospect our expert overall rating is 0, but the original results are provided here. ^t^This study validated rs-fMRI SOZ to that of SEEG findings in DRE from multiple heterotopic nodules, thus the patient selection does match the study question by expert review, though the assigned reviewers rated it otherwise. The rs-fMRI connectivity between the heterotopias corresponded to that found by seizure and epileptiform activity by SEEG. Thus, while the two reviewers who evaluated this specific study in 28 consecutive patients were concerned if the comparison lined up with the study question, the expert review consensus is rated at 0, though again the original finding are provided.

### 2.5 Statistical methods

The odds ratio was used to quantify the agreement between rs-fMRI and the comparative modalities. An odds ratio larger and less than 1 implies agreement and disagreement, respectively, while an odds ratio of 1 implies that the modalities are independent. Two types of models were used, namely, fixed-effects models and random-effects models (Borenstein et al., 2010). Differences in odd ratios between studies are viewed as systematic differences in fixed-effects models but as random variation in random-effects models.

#### 2.5.1 Fixed-effects analysis

Each study was summarized into a 2×2 table. In the fixed-effects analysis, odds ratios that quantify the agreement between rs-fMRI and the comparative, with exact confidence intervals (CIs), were estimated based on the conditional likelihood. Only 10 studies that had non-zero margins were used in the fixed-effects analysis. In five studies, one cell was zero, and median unbiased estimates were obtained. The heterogeneity of the odd ratios was tested using the likelihood ratio test. As this test requires a large sample in each study, it was restricted to the seven studies with at least 20 subjects and no zero margins (29 to 64 patients per study, 298 patients in total). Sensitivities and specificities from the individual studies were calculated with their respective 95% CI intervals.

Among the 7 studies with at least 20 subjects and no zero margins, differences in agreement with rs-fMRI across modalities were evaluated using a four-level categorization of modality: Anatomical MRI, EEG, iEEG, and surgical outcome. Differences in agreement with rs-fMRI between modalities (three comparisons) and between studies within modalities (three comparisons) were then evaluated. Differences between and within modalities were evaluated using a likelihood ratio test, and significance was evaluated using a *p*-value threshold of 0.05.

#### 2.5.2 Random-effects analysis

The random-effects model included all studies (*n* = 25) and was a generalized mixed model with three independent random effects per study: one for each marginal log odds (rs-fMRI and comparative) and one for the log odds ratio. The predicted study-specific odds ratios and their respective 95% prediction intervals (PI) were computed. The heterogeneity of odd ratios was tested using the likelihood ratio test. Sensitivities and specificities from the individual studies were calculated with their respective 95% PI intervals. SAS software version 9.4/15.2 was used. Data and code are available in an open repository.^2^

## 3 Results

### 3.1 Study characteristics

**Figure 1:**
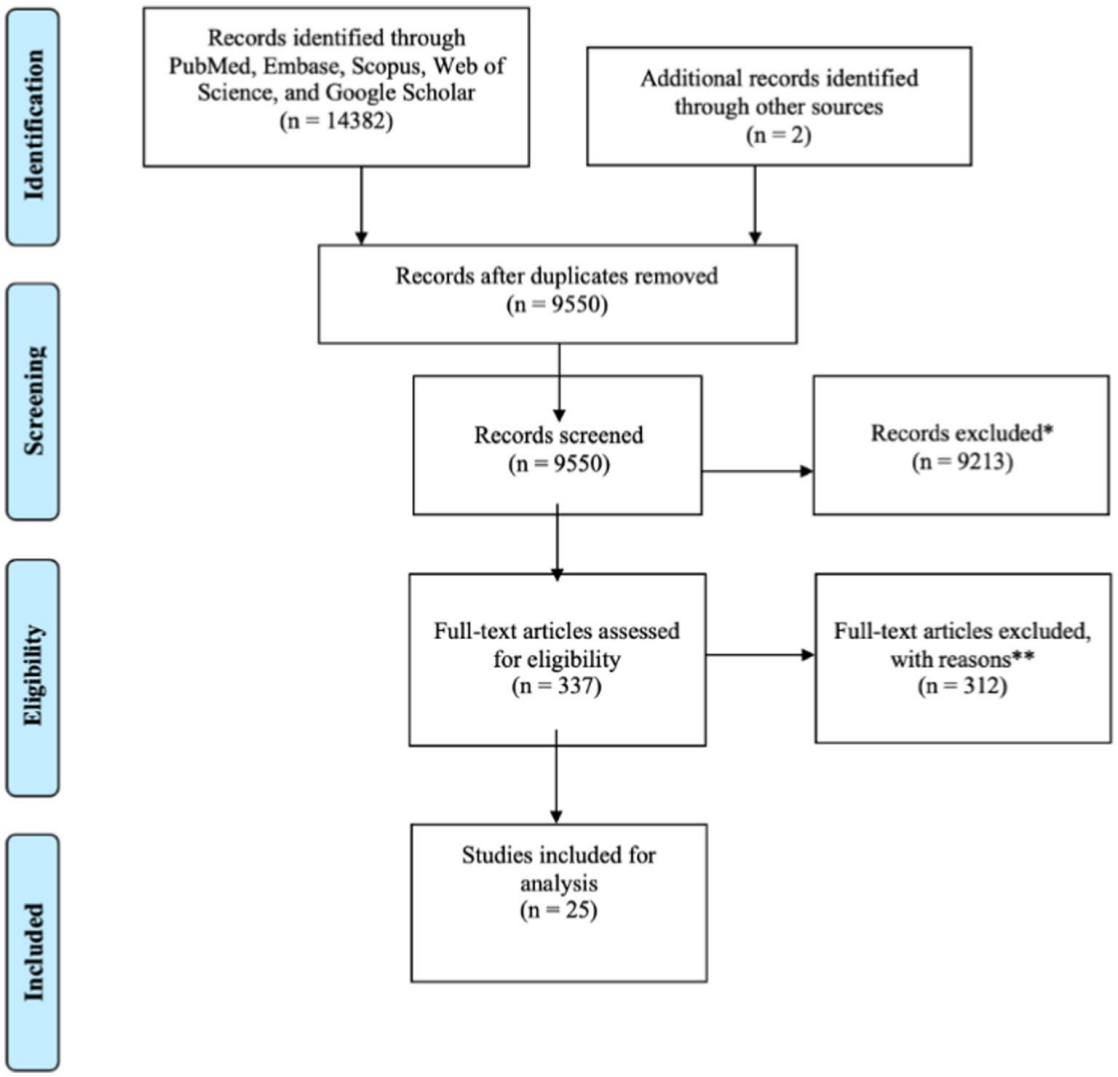
PRISMA flow diagram for the current meta-analysis. *Animal studies, patients without epilepsy; **study design (*n* = 141), indication (*n* = 25), comparator (*n* = 12), publication type (*n* = 20), intervention (*n* = 9), outcomes (*n* = 9), patient population (*n* = 4), setting (*n* = 3), language (*n* = 4), manuscript unavailable (*n* = 11), duplicate paper (*n* = 8), duplicate population (*n* = 14), and unavailable subject level SOZ data (*n* = 52).

The systematic literature review resulted in 25 studies qualifying for inclusion (Figure 1). Reasons and quantities for study exclusion are delineated in Figure 1. Of the three Hunyadi et al. (2014, 2015a, 2015b) publications, there is potential for unconfirmed population overlap. However, there is clear evidence of unique participant data as the comparative modalities used in each were different (e.g., EEG, EEG-fMRI, and surgical outcome). Based on these distinctions, we concluded that the subjects were unique across studies.

The inter-rater reliability data of the reviewers’ evaluation of the abstract of the publications are provided in Table 4. Inter-rater reliability of the publications reflected overall good agreement between raters, with proportionate agreement between 0.7 and 1 for all but one rater pairing at 0.33, and random agreement probability between 0.88–1.0 for all but two rater pairings at 0.55 (Table 4). Cohen’s Kappa coefficient range was −0.5 to 0.47, which is low. However, this ratio’s value was indeterminate in six rater pairings; thus, proportionate agreement was determined to be more informative of the study findings.

**Table 4:** Inter-rater reliability of abstract screening.

Reviewer A	Reviewer B	A Yes, B Yes*	A Yes, B No**	A No, B Yes***	A No, B No*	Proportionate Agreement	Yes Probability	No Probability	Random Agreement Probability	Cohen’s Kappa
Reviewer 3	Reviewer 1	9	12	54	1,209	0.949	0.001	0.935	0.936	0.195
Reviewer 6	Reviewer 7	5	0	47	460	0.908	0.001	0.890	0.891	0.161
Reviewer 5	Reviewer 1	29	12	58	865	0.927	0.004	0.871	0.875	0.420
Reviewer 1	Reviewer 4	104	158	39	357	0.701	0.087	0.471	0.558	0.323
Reviewer 6	Reviewer 3	0	7	3	948	0.990	0.000	0.990	0.990	−0.004
Reviewer 2	Reviewer 1	27	52	29	1,442	0.948	0.002	0.915	0.917	0.374
Reviewer 6	Reviewer 4	17	18	32	647	0.930	0.003	0.886	0.889	0.369
Reviewer 3	Reviewer 7	0	1	15	145	0.901	0.001	0.901	0.902	0.012
Reviewer 6	Reviewer 5	0	5	1	439	0.987	0.000	0.987	0.987	0.004
Reviewer 6	Reviewer 1	24	35	23	847	0.938	0.003	0.890	0.892	0.420
Reviewer 5	Reviewer 3	1	2	3	312	0.984	0.000	0.978	0.978	0.278
Reviewer 6	Reviewer 9	2	2	2	69	0.947	0.003	0.896	0.899	0.472
Reviewer 8	Reviewer 6	3	14	0	154	0.918	0.002	0.885	0.887	0.279
Reviewer 5	Reviewer 4	1	2	3	132	0.964	0.001	0.950	0.951	0.268
Reviewer 5	Reviewer 2	0	0	4	128	0.970	0.000	0.970	0.970	0.000
Reviewer 6	Reviewer 2	0	0	0	15	1.000	0.000	1.000	1.000	NaN
Reviewer 3	Reviewer 4	2	0	6	230	0.975	0.000	0.959	0.959	0.392
Reviewer 7	Reviewer 4	1	4	0	21	0.846	0.007	0.777	0.784	0.288
Reviewer 5	Reviewer 7	0	0	3	32	0.914	0.000	0.914	0.914	0.000
Reviewer 3	Reviewer 2	1	1	2	73	0.961	0.0010	0.936	0.937	0.381
Reviewer 9	Reviewer 4	0	1	1	1	0.333	0.111	0.444	0.556	−0.500
Reviewer 7	Reviewer 1	0	0	1	20	0.952	0.000	0.952	0.952	0.000
Reviewer 8	Reviewer 1	0	1	1	35	0.946	0.001	0.947	0.947	0.028
Reviewer 9	Reviewer 5	0	0	0	12	1.000	0.000	1.000	1.000	NaN
Reviewer 8	Reviewer 3	0	0	0	17	1.000	0.000	1.000	1.000	NaN
Reviewer 9	Reviewer 7	0	0	0	8	1.000	0.000	1.000	1.000	NaN
Reviewer 8	Reviewer 7	0	1	0	15	0.938	0.000	0.938	0.938	0.000
Reviewer 2	Reviewer 4	0	0	0	3	1.000	0.000	1.000	1.000	NaN
Reviewer 9	Reviewer 3	0	2	0	19	0.905	0.000	0.905	0.905	0.000
Reviewer 8	Reviewer 5	0	0	0	6	1.000	0.000	1.000	1.000	NaN
Reviewer 8	Reviewer 4	0	0	1	2	0.667	0.000	0.667	0.667	0.000
Reviewer 9	Reviewer 1	0	0	0	1	1.000	0.000	1.000	1.000	NaN

*“A Yes, B Yes” and “A No, B No” signify the number of articles that both interviewers voted “Yes” or “No” on, respectively. **“A Yes, B No” signifies the number of articles that Reviewer A voted “Yes” and Reviewer B voted “No.” ***“A No, B Yes” signifies the number of articles that Reviewer A voted “No” and Reviewer B voted “Yes.” NaN, not a number or estimable.

Of the 337 publications with full-text review, 312 were excluded, with the most common reason being study design (141) not matching with the study question, which required a known comparator on a 1:1 basis for the rs-fMRI SOZ.

The most common rs-fMRI analysis methods were ICA and SBC, utilized in nine and eight studies, respectively. For studies not meeting inclusion criteria, the analysis methods are in Table 5. The analyses were categorized as “data-driven” if the whole brain was investigated and “hypothesis-driven” if the study investigated a reduced set of regions of interest. The comparative modalities included aMRI, EEG, iEEG, EEG-fMRI, MEG, and surgical outcomes.

**Table 5:** rs-fMRI analysis methods from all evaluated publications.

Analysis methods from studies included in meta-analysis
Amplitude of low frequency fluctuation/fractional ALFF
General linear mode
Global signal regression
Hemodynamic response function
Independent component analysis
Intrinsic connectivity contrast
Principal component analysis
Regional homogeneity
Temporal clustering analysis
Analysis methods from studies excluded from meta-analysis
Bootstrap analysis of stable clusters
Detection of abnormal networks in individuals
Four-dimensional consistency of local neural activities analysis
Functional connectivity density
Gaussian random field theory
Graph theory
Time shift voxel mirrored homotropic connectivity

Various rs-fMRI analysis methods used in studies that were both included and excluded from the meta-analysis.

From the remaining 25 included studies, 452 rs-fMRI SOZ patient comparisons were evaluated. The age range was 18 months to 66 years old (Table 6). Six studies included children: Lee et al. (2014), Reyes et al. (2016), Boerwinkle et al. (2017), Chen et al. (2017), Boerwinkle et al. (2019), and Zhao et al. (2019).

**Table 6:** Demographics, rs-fMRI software, and comparative modality used in studies included in the meta-analysis.

Study	*n*	Age Mean (range)	% Female	Comparative modality	Software; analysis method;	Data vs. Hypothesis	Rs-fMRI agreed with modality	Findings summary
Anzellotti et al. (2010)	1	40	100	MEG	Brain Voyager QX; ICA;	data	Yes	From MEG data an epileptic focus was localized in the left posterior insular gyrus (LPIG). FMRI data evidenced that sexual excitation symptoms with PGAD could be correlated with an increased functional connectivity (FC) between different brain areas: LPIG (epileptic focus), left middle frontal gyrus, left inferior and superior temporal gyrus and left inferior parietal lobe. The reduction of the FC observed after antiepileptic therapy was more marked in the left than in the right hemisphere in agreement with the lateralization identified by MEG results. Treatment completely abolished (seizure) symptoms and functional hyperconnectivity.
Song et al. (2006)	2	NR	NR	aMRI	SPM; ICA, PCA*; data	data	Yes	It has shown that the results derived from the application of our algorithm to vivo glioma fMRI data are conformed to those determined by the presurgical assessments
Wang et al. (2007)	2	24.5 (20–29)	50	aMRI	SPM; SBC;	data	Yes	These identified regions by rs-fMRI are consistent with the results of clinical MRI.
Tavares et al. (2017)	3	40 (19–60)	33	EEG	MB DPARSF, SPM; GLM, SBC*	data	Yes	FC is able to detect the brain regions associated with epileptogenic tissue.
Weaver et al. (2013)	4	37.8 (34–44)	50	iEEG	MB, FSL; ReHo	data	Yes	At the group level, there was decrease in the rank for ROI harboring the seizure focus for the ReHo rankings as well as for the mean rank. At the individual level, the seizure focus ReHo rank was within bottom 10% lowest ranked ROIs for all epilepsy patients and three out of the four for the IRC rankings. However, when the two ranks were combined (averaging across ReHo and IRC ranks and scalars), the seizure focus ROI was either the lowest or second lowest ranked ROI for three out of the four epilepsy subjects. This suggests that rsfMRI may serve as an adjunct pre-surgical tool, facilitating the identification of the seizure focus in focal epilepsy.
Gnanadas et al. (2017)	6	NR	NR	iEEG	NR; ReHo, f/ALFF*	data	Yes	The ReHo result found that the seizure identified is in the same region as described by IEEG.
Morgan et al. (2004)	6	42 (29–63)	83	surgical	ML, SPM, IDL; TCA	hypo	Yes	In all six patients who underwent respective surgery, the fMRI with temporal clustering analysis accurately determined the epileptogenic hippocampal hemisphere (*p* = 0.005). In the three subjects without confirmed localization, the technique determined regions of activity consistent with those determined by the presurgical assessments.
Stufflebeam et al. (2011)	6	NR (18–24)	33	iEEG	NR; SBC	hypo	Yes	The foci identified by FC imaging overlapped the epileptogenic areas identified by iEEG in all 5 patients.
Zhao et al. (2019)	6	25.2 (13–43)	66	EEG	MB, freesurfer; SBC	hypo	Yes	This study compared the cortical presence of abnormal discharges at SOZ, recorded by SEEG, with the interictal spatial correlation patterns of spontaneous BOLD fluctuations. The consistency between these two modalities was proved by a high AUC in ROC curve.
van Houdt et al. (2015)	7	31.6 (22–48)	28	surgical	FSL MELODIC, SPM; ICA	data	Yes	The epilepsy-related rs-fMRI ICs selected from the fMRI data with IEDs and from the data without IEDs for all patients. In general the ICs that were selected as epilepsy- related show good resemblance with the resection masks and EEG–fMRI correlation patterns.
Jann et al. (2008)	8	42.8 (19–66)	25	EEG	BrainVoyager QX; ICA	data	Yes	ICA EEG/fMRI can be used to identify regions with hemodynamic changes during epileptic activity that reflect the irritative zone, resembling, at least in part, the epileptogenic zone.
Kang et al. (2003)	8	NR	NR	EEG-fMRI	MB fmristat: HRF	data	Yes	The activated areas obtained with the patient-specific HRFs were larger or similar to the originally activated areas. Additional activated areas were seen in five patients, and most were compatible with the EEG and anatomical MRI localization of epileptogenic and lesional regions.
Hunyadi et al. (2014)	10	NR	NR	EEG-fMRI	SPM, FSL (Fix); ICA	data	Yes	In 10 focal epilepsy patients and 13 healthy control, FC reached 77% specificity, indicating that the proposed technique reliably selects ICs related to epileptic activity.
Yang et al. (2015)	11	35.9 (22–56)	66	aMRI	MB DPARSF, REST; f/ALFF	data	Yes	Lateralization of TLE based on FC. Laterality encoded in intra- regional, inter-regional, and whole brain FC to achieve 83% correct rate on a small cohort. RF-based feature selection, along with relative feature importance analysis, provides a multivariate analysis method for lateralization.
Hunyadi et al. (2015b)	12	NR	NR	EEG	SPM, FSL MELODIC; ICA	data	Yes	The GLM activation map showed a single activation cluster overlapping with the EZ in 3 out of 12 cases. In 6 cases it showed activation both in the EZ and in one or more remote areas. Finally, in 3 cases no significant activation was present.
Hunyadi et al. (2015a)	18	NR	NR	surgical	SPM, FSL (Fix); ICA	data	Yes	FC correctly indicates the EZ in several (*N* = 4) EEG-negative cases but at the same time maintaining a high specificity (92%). fMRI can be used in a prospective manner, extending to EEG-negative cases.
Su et al. (2015)	21	28.5 (18–43)	57	EEG	SPM; SBC	hypo	Yes	Discriminative analysis of FC indicated patients with mesial temporal lobe epilepsy with right hippocampal sclerosis exhibited decreased FC within the right hemisphere and increased FC within the left hemisphere. FC typically obey the hemispheric lateralization trend and most of the functional connections that disturb the lateralization trend are the intranetwork ones.
Barron et al. (2015)	23	NR	56	surgical	MB, FSL, freesurfer; SBC	hypo	Yes	This study shows that thalamic functional connections are sensitive and specific markers of seizure onset laterality in individual temporal lobe epilepsy patients.
Lee et al. (2014)	29	29.4 (7–55)	51	iEEG	NR; ICA	data	Yes	FC measurement using rs-fMRI has the potential to provide a novel noninvasive method to localize the SOZ as a part of presurgical evaluation. The concordance rate between the icEEG SOZ and fMRI-ICC map was better in patients with good outcome, suggesting that this kind of approach can be useful as a biomarker that reflects epileptogenesis and predicts surgical outcome in advance when planning epilepsy surgery.
Reyes et al. (2016)	34	36.1 (16–66)	47	EEG	MB DPARSF, SPM; GSR, f/ALFF	hypo	Yes	We found that fALFF, a resting-state fMRI measure of regional BOLD signal, is sensitive and specific to focal pathology in TLE.
Boerwinkle et al. (2017)	36	12.3 (1–19)	42	surgical	FSL MELODIC; ICA	data	Yes	In the RS group, the EZ target within the (surgical target) was ablated with high accuracy (>87.5% of target ablated in 83% of subjects). There was no difference between the groups in percentage of ablated volume (*p* = 0.137). Overall seizure reduction was higher in the rs‐fMRI group: 85% RS versus 49% non-rs-fMRI targeted (CS) (*p* = 0.0006, adjusted). The Engel outcomes showed differences in those with freedom from disabling seizures (class I), 92% RS versus 47% CS, a 45% improvement (*p* = 0.001). Compared to prior studies, there was improvement in class I outcomes (92% vs. 76–81%).
Chen et al. (2017)	42	24 (5–50)	42	aMRI	SPM; f/ALFF	data	Yes	RS-fMRI showed comparable sensitivity to PET (83.3%) and specificity to VEEG (66.7%), respectively, for EZ localization in patients with focal epilepsy. There were no significant differences between RS-fMRI and the other localization techniques in terms of sensitivity, specificity, PPV, and NPV. The sensitivities of ReHo, ALFF, and fALFF were 69.4, 52.8, and 38.9%, respectively, and for specificities of 66.7, 83.3, and 66.7%, respectively. There were no significant differences among ReHo, ALFF, and fALFF, except that ReHo was more sensitive than fALFF.
Bettus et al. (2010)	44	38.5 (20–61)	54	EEG	SPM; SBC	hypo	Yes	Basal functional connectivity (BFC) decreases were found bilaterally, although the number of decreased links was significantly higher in the epileptogenic side (*p* = 0.025). Conversely, BFC increases were found almost exclusively in the contralateral lobe leading to a strong test effect for locating the non-epileptic lobe with a sensitivity of 64% and a specificity of 91% (*p* < 0.001).
Khoo et al. (2019)	49	27.6 (19–52)	56	iEEG	MB, SPM, CONN; SBC	hypo	Yes	We found that fALFF, a resting-state fMRI measure of regional BOLD signal, is sensitive and specific to focal pathology in TLE.
Boerwinkle et al. (2019)	64	9.8 (2–21)	42	surgical	FSL MELODIC; ICA	data	Yes	Network‐targeted surgery, followed by postoperative rs‐fMRI normalization was significantly (*p* < 0.001) correlated with seizure reduction, with a Spearman rank correlation coefficient of 0.83. Of 39 cases with postoperative rs‐fMRI SOZ normalization, 38 (97%) became completely seizure free. In contrast, of the 25 cases without complete rs‐fMRI SOZ normalization, only 3 (5%) became seizure free. The accuracy of rs‐fMRI as a biomarker predicting seizure freedom is 94%, with 96% sensitivity and 93% specificity.

*All participants received both; data-driven – the whole brain was queried; hypothesis-driven – a reduced set of locations was investigated based on *a priori* knowledge or hypothesis. hypothes, hypothesis; ML, MATLAB; surgical, surgical outcome; aMRI, anatomical MRI; SBC, seed-based correlation; DPARSF, Data Processing Assistant for Resting-State; ALFF, amplitude of low frequency fluctuation; fALFF, fractional ALFF; F, female; FSL, FMRIB Software Library; GLM, General Linear Model; GSR, Global Signal Regression; HRF, Hemodynamic Response Function; ICA, Independent Component Analysis; ICC, Intrinsic Connectivity Contrast; IDL, Interactive Data Language; MELODIC, Multivariate Exploratory Linear Optimized Decomposition into Independent Components; NR, Not Reported; PCA, Principal Component Analysis; ReHo, Regional Homogeneity; REST, Resting-State fMRI Data Analysis Toolkit; SPM, Statistical Parametric Mapping; TCA, Temporal Clustering Analysis.

Of the studies utilizing imaging modalities to define SOZ truth, two counted participants more than once (which is well accepted given the method explanation): Bettus et al. (2010) and Khoo et al. (2019). Bettus et al. (2010) evaluated the left and right hemisphere separately. Khoo et al. (2019) evaluated heterotopic nodular pairs within patients separately. Of the studies utilizing surgical outcomes to define ground truth, each surgery counted as a separate comparison event for corresponding pre-operative rs-fMRI (Boerwinkle et al., 2019). They investigated 64 surgical outcomes among 58 participants, four participants had two surgeries, and one participant had three surgeries.

### 3.2 Assessment of bias

Per the QUADAS-2 Bias Assessment Tool, there are four domains of bias: patient selection, index test, reference test, and flow and timing (Whiting et al., 2011), with results as delineated in Table 3. According to QUADAS-2 guidelines, to qualify for downgrading evidence, a publication must have two domains with high-risk bias. None of the included publications had bias meeting this level of concern. Further, of the areas rated as high risk, two were overturned by further expert review as described below.

For the patient selection domain, seven studies were rated as high risk due to selection of patients that was not well specified, such as being in consecutive order or provided reasoning otherwise. One had concern for population type (Whiting et al., 2011). This study validated rs-fMRI SOZ to that of SEEG in DRE due to multiple heterotopic nodules. Thus, the patient selection does match the study question by expert review, though the assigned reviewers rated it otherwise. The rs-fMRI connectivity between the heterotopias corresponded to that found by seizure and epileptiform activity by SEEG. Thus, while the two reviewers who evaluated this specific study in 28 consecutive patients were concerned if the comparison lined up with the study question, the expert review consensus is rated as low risk, although again the original finding is provided.

In the reference test domain, one study was of high risk (Whiting et al., 2011). In this study, six patients who underwent SEEG and rs-fMRI were analyzed by predefined metrics. Thus, it is not clear why our interpreters rated this study as possibly biased since the methods removed expert opinion from interpretation. Because of this, in retrospect, our expert overall rating is no concern, although the original results are provided in the table.

### 3.3 Assessment of agreement

#### 3.3.1 Descriptive results

Of the 25 studies included in the study, 9 had a sample size of 20 or more subjects. While 10 had no zero margins, 6 had one zero margin, and 9 had two zero margins. Only 7 studies had a sample size over 20 and no zero margins. A single zero margin indicates that either one row or one column total is zero as presented in Table 1. The presence of two zero margins implies that only one of the four cell counts was not zero. For example, Anzellotti et al. (2010) had a sample size of one, resulting in only one non-zero cell. Thus, the number of studies with the respective comparative modalities was EEG 7, surgical outcome 6, iEEG 5, aMRI 4, EEG-fMRI 2, and MEG 1. Of the 9 studies with greater than 20 subjects, patients were more often in the adult than pediatric age range, and 5 studies used the SPM software for analysis, whereas 3 studies used FSL; and, of these, 5 took a hypothesis-based approach (meaning evaluating only certain regions of interest), whereas 4 were data-driven (meaning evaluating the entire brain).

#### 3.3.2 Fixed-effects analysis

**Figure 2:**
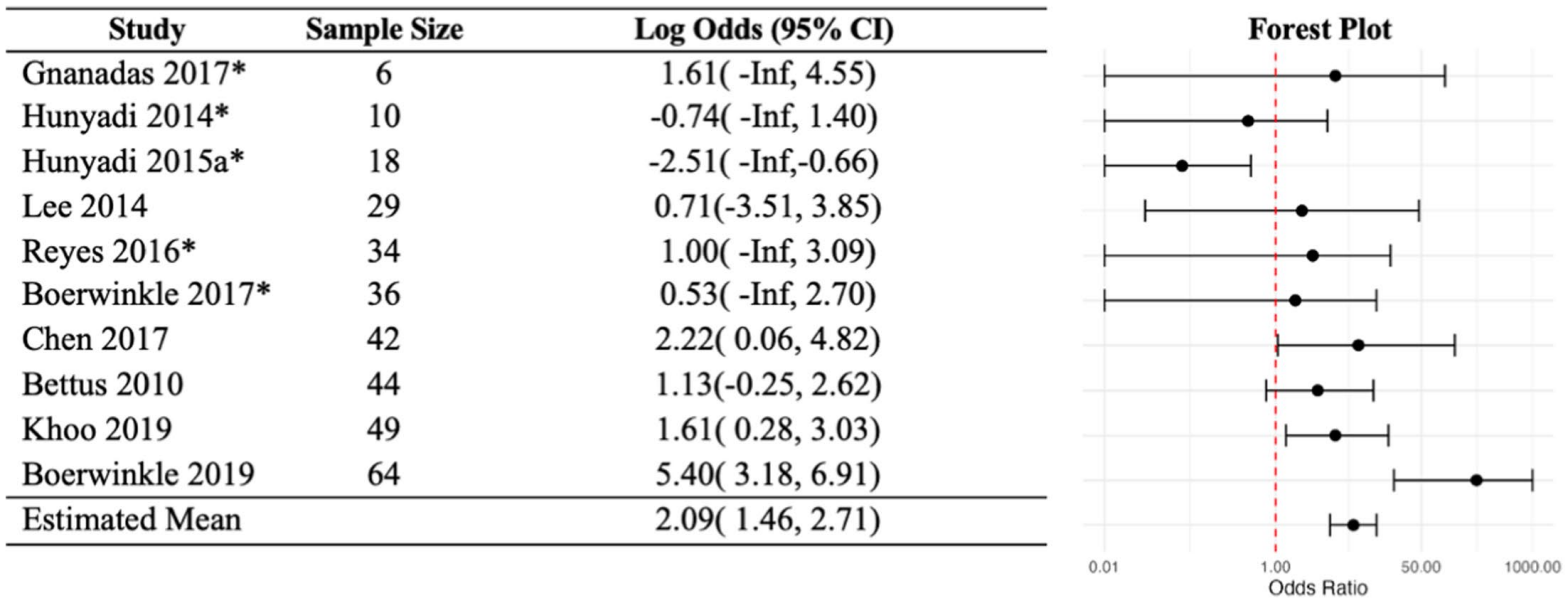
Fixed-effects analysis odd ratios. Each study was summarized into a 2×2 table, and conditional likelihood-based estimates were obtained if there are no zero cells. In five studies, one cell was zero, and median unbiased estimates were obtained (as marked by *). If any margin is zero, the study is non-informative. This left the 10 studies shown in this figure. The analysis presented very strong evidence against the existence of a common odds ratio. Red line indicates an odds ratio of 1 of no association.

**Figure 3:**
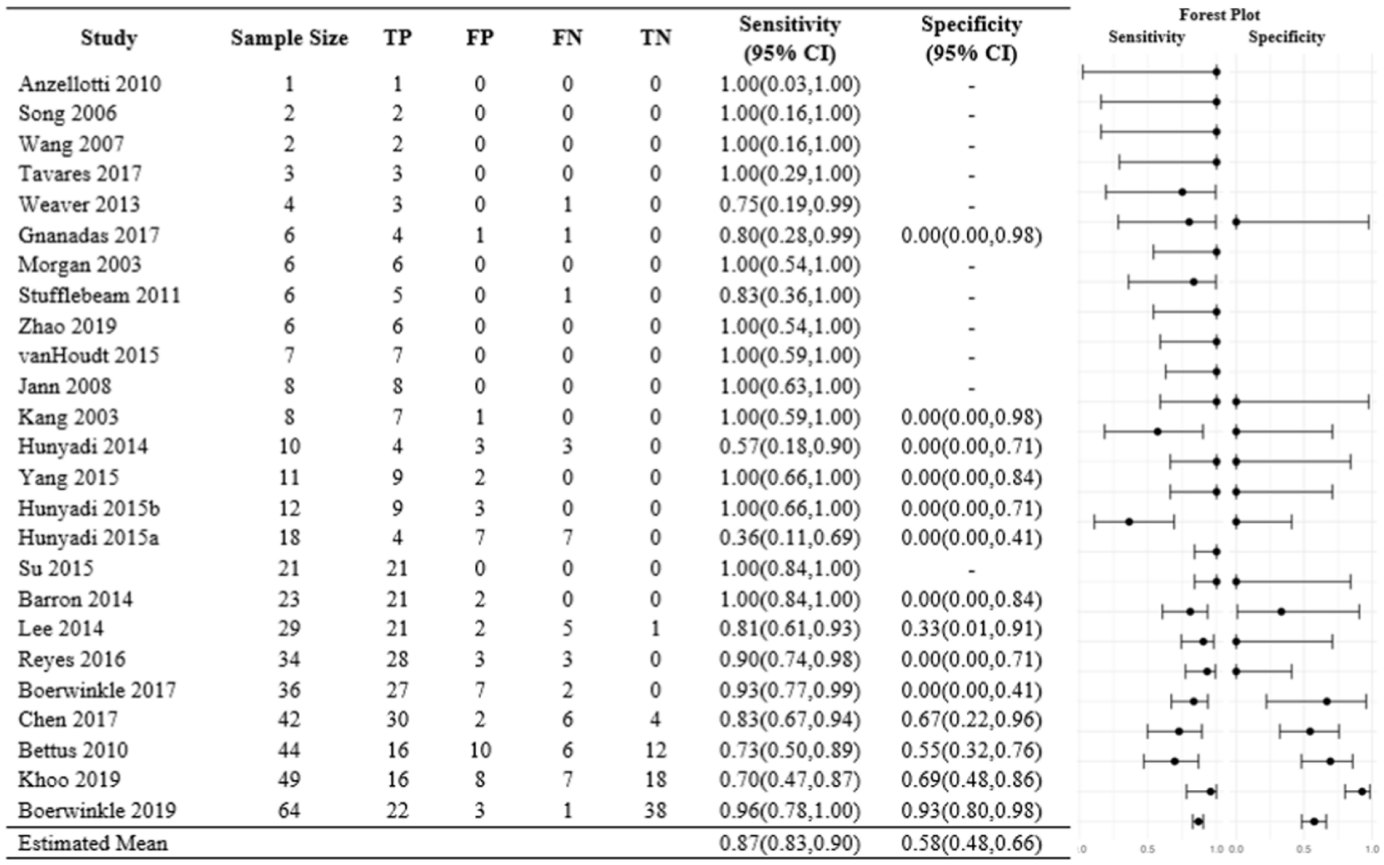
Fixed-effects analysis sensitivities and specificities. Each study was summarized into a 2×2 table. Sensitivities and specificities were calculated, and exact 95% confidence intervals were obtained.

In the fixed-effects analysis, the odds ratio estimates for the 10 studies with no zero margins ranged between 0.08 and 222 (Figure 2). Only three studies have 95% CI above 1. Only one study, Hunyadi et al. (2015a), had an interval below 1 indicating disagreement between rs-fMRI SOZ and the comparative modality. The heterogeneity of the odds ratios was significant indicating high evidence in the favor of heterogeneity (*p* < 0.001). The estimated common odds ratio is 8.0 with a 95% confidence interval of 4.31 and 14.90. However, the usefulness of this single estimate is highly questionable because there is strong evidence against the existence of a common odds ratio. The overall sensitivity was 0.87, and the study estimates varied between 0.36 to 1.00 (Figure 3). In comparison, the overall specificity was 0.58, and the study estimates varied between 0.00 and 0.92 among studies with no-zero margins. Estimated specificities were always lower than sensitivities. Boerwinkle et al. (2019) had both a high sensitivity (0.96) and specificity (0.93) estimate.

Among the seven studies with no-zero margins and sample size above 20, the counts of anatomical MRI, EEG, iEEG, and surgical outcome were 1, 2, 2, and 2, respectively (total: 298 subjects). Positive agreement with rs-fMRI was observed with anatomical rs-fMRI (*p*-val = 0.04), iEEG (*p*-val = 0.01), and surgical outcome (*p*-val < 0.001) (Table 7). The fixed-effects analysis showed marked differences in agreement with rs-fMRI between modalities (*p* = 0.005), as well as differences in agreement between studies using the same modality (*p* = 0.002). That is, there is strong evidence of differences in agreement with rs-fMRI across the four modalities and across studies within each modality. Moreover, the level of agreement between rs-fMRI and the comparative was highest for surgical outcome with an odds ratio of 48.0 (95% CI: 10.4–334), higher than agreement between rs-fMRI and EEG (*p* = 0.002) and between rs-fMRI and iEEG (*p* = 0.007), but not significantly different from agreement between rs-fMRI and anatomical MRI (*p* = 0.173). The considerable difference in estimated agreement between Boerwinkle et al. (2017) (OR 1.7) and Boerwinkle et al. (2019) (OR 222), two studies sharing the same modality, highlights the heterogeneity of agreement between studies within each modality (Table 7). This suggests the possibility of experience level increasing expert-derived SOZ and post-operative seizure outcome concordance.

**Table 7:** Fixed effects analysis of agreement heterogeneity within and between modalities (7 Studies, 298 subjects).

Comparative modality and study	Odd ratio (95% CI)	*p*-value
Anatomical MRI	9.24 (1.06, 124.40)	0.043
Chen et al. (2017)	9.24 (1.06, 124.40)	0.043
EEG	2.51 (0.69, 9.32)	0.1888
Reyes et al. (2016)*	2.72 (<0.01, 21.86)	0.751
Bettus et al. (2010)	3.11 (0.78, 13.78)	0.124
iEEG	4.18 (1.29, 14.36)	0.014
Lee et al. (2014)	2.04 (0.03, 47.12)	1.000
Khoo et al. (2019)	4.96 (1.32, 20.83)	0.015
Surgical outcome	48.00 (10.38, 334.19)	<0.001
Boerwinkle et al. (2017)*	1.70 (<0.01, 14.93)	0.644
Boerwinkle et al. (2019)	221.74 (24.01, >100.00)	<0.001

Each study was summarized into a 2×2 table. Seven studies with at least 20 subjects and no zero margins were used to fit a log-linear model to estimate the odd ratios between rs-fMRI and each comparative modality with their respective 95% exact confidence intervals. The *p*-values correspond to the hypothesis of no association between rs-fMRI and the respective modality. In two studies, one cell was zero leading to the calculation of median unbiased estimates where the *p*-value represents a one-sided test (as marked by *). Odd ratio estimates for Chen et al. (2017) are equivalent to the overall estimate for Anatomical MRI as it was the only study included within the modality category. Odd Ratios and *p*-values were calculated for 7 studies to evaluate agreement heterogeneity of modalities.

#### 3.3.3 Random-effects analysis

**Figure 4:**
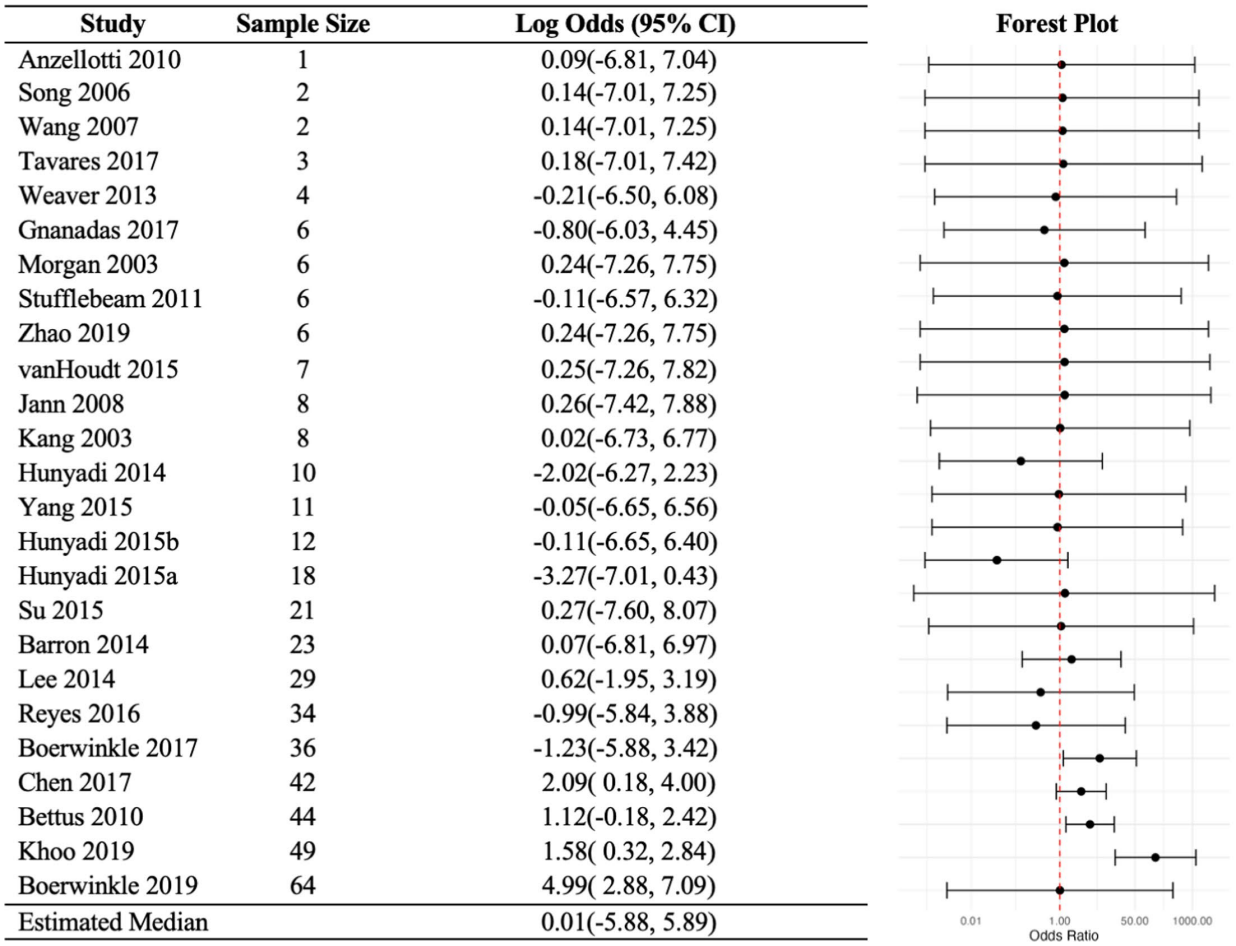
Random-effects analysis odd ratios. Random-effects model includes a random study-specific log odds ratio measuring the agreement between fs-MRI and the comparative. Predicted study-specific odds ratios and prediction intervals are depicted. The study-specific odds ratios, assumed normally distributed, are estimated to have a mean (and median) of 1.007 and with a standard deviation of 3. The analysis presented very strong evidence against the existence of a common odds ratio.

For the random-effects analysis, the population median odds ratio was estimated to be 1.007 (95% PI: 0.003–361.41), and the predicted odd ratio estimates for all 25 studies ranged from 0.04 to 147 (Figure 4). As in the fixed-effects analysis, the random-effects analysis identified the same three studies with a 95% PI above 1. In addition, it is estimated that, in the underlying population of studies, the log odds ratio is centered approximately 0.0069 (95% CI: −2.59 to 2.60) with a standard deviation of 3. This implies, for example, that the population quartiles of log odds ratio are −2 and + 2, a 54-fold increase in the agreement odds ratio going from the first to the third quartile which is indicative of considerable between-study heterogeneity. The null hypothesis of no population heterogeneity among the odds ratios is rejected (*p* = 0.001). These results suggest that the agreement between rs-fMRI and comparative modalities varies widely across studies. Most studies resulted in odds ratios close to 1 in both the fixed-effects and random-effects analyses. However, Boerwinkle et al. (2019) stood out with the largest estimated odds ratios, with values of 222 in the fixed-effects analysis and 148 in the random-effects analysis.

**Figure 5:**
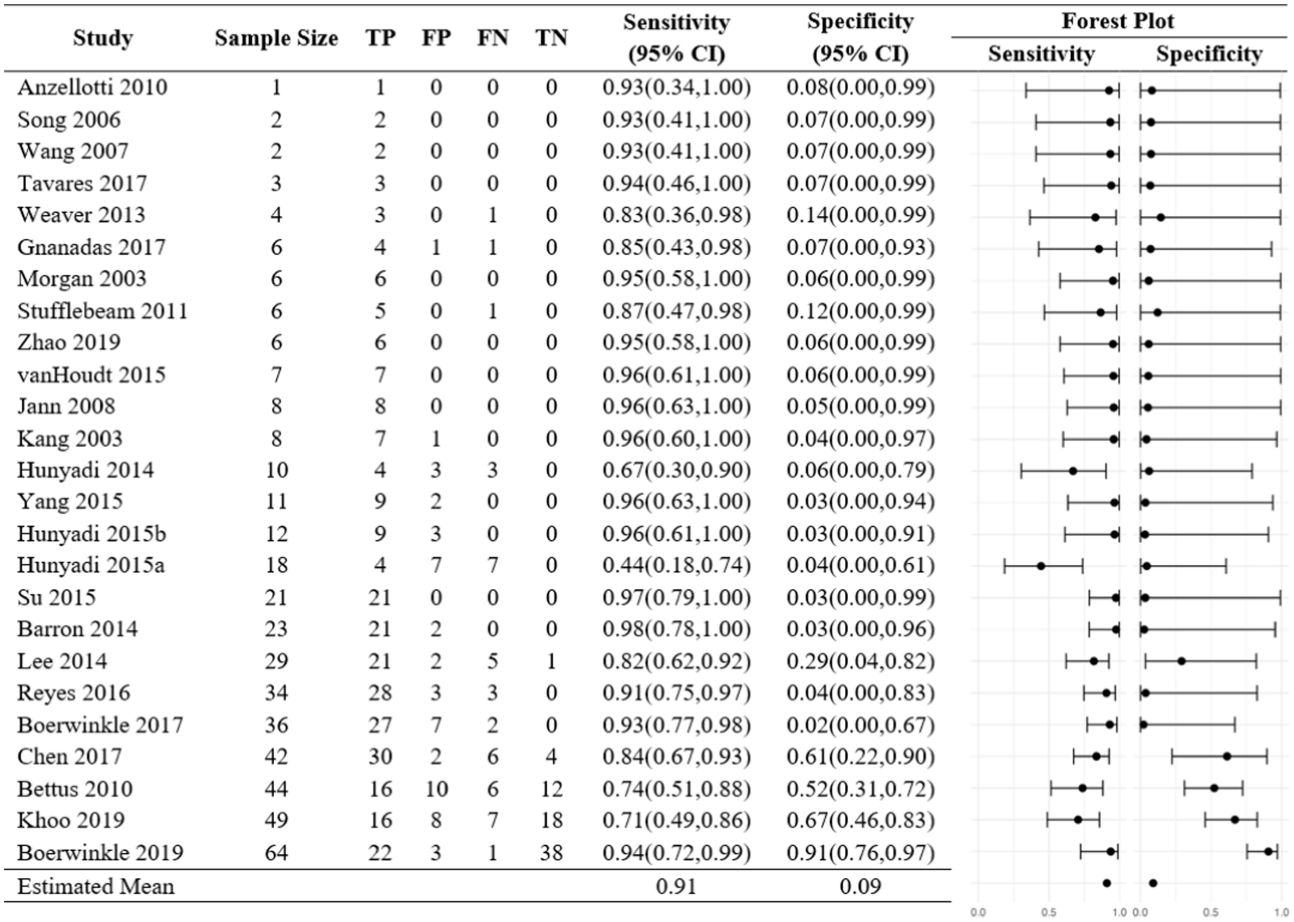
Random-effects sensitivities and specificities. Random-effects model includes a random study-specific log odds ratio measuring the agreement between fs-MRI and the comparative. Predicted study-specific odds ratios and prediction intervals are depicted. The study-specific sensitivity and specificity assumed normality are assumed to have a mean of 0.91 and 0.09, respectively.

The median probability of a positive rs-fMRI is 0.91, and 95% of studies fall between 0.33 and 0.995. The analogous estimates for the comparative are 0.87 and 0.38 to 0.985. That is, rs-fMRI and the comparative have similar levels of positive detection. It is worth noting that this similarity has no bearing on agreement or disagreement. Moreover, the population mean sensitivity is 0.91 and the predicted sensitivities for all 25 studies ranged between 0.44 and 0.96. The population mean for specificity is lower at 0.09, and the predicted values varied, ranging between 0.02 to 0.67 (Figure 5). Most studies had large sensitivities and low specificity estimates with wide prediction intervals.

## 4 Discussion

We expanded upon the prior meta-analysis of rs-fMRI SOZ by ICA/PCA validated by surgical outcomes or iEEG that found a common odds ratio of 2.63 (95% CI: 0.66–10.6) (Nagahama et al., 2018), by removing all restrictions and characterizing the broader library of rs-fMRI SOZ analysis and extent of validation modality subtypes. We evaluated overall agreement and then investigated whether this agreement differed between modality groups and within studies under each modality. The overall evaluation was geared to help us understand what the effect size of rs-fMRI agreement was overall regardless of comparator, the between modality evaluation could help identify if and which types of modalities had the highest agreement with rs-fMRI, and the within modality evaluation would help us understand if significant differences in agreement between studies sharing the same modality were present. In addition, we tested the heterogeneity of odd ratios to evaluate the validity of a common odds ratio to describe overall agreement of SOZ localization between rs-fMRI and the comparators across studies.

### 4.1 Evaluation of agreement

The fixed-effects analysis presented some indicators of SOZ agreement. (1) The study-specific common odds ratio was estimated to be 8.00 (95% CI: 4.3–14.9). Nonetheless, the usefulness of this single estimate is highly questionable because there is strong evidence against the existence of a common odds ratio (*p* < 0.001). (2) Out of the 10 studies with no zero margins included in the fixed-effects analysis, 8 studies estimated positive agreement, and 2 studies estimated negative agreement. (3) In the 4 largest studies, those with greater than 40 subjects, the odd ratio estimates, and the respective 95% confidence intervals were above 1. (4) Only one of the 25 included studies showed poor agreement between rs-fMRI and a comparator. Hunyadi et al. (2015a) found that rs-fMRI localized the SOZ to a different location than their comparative modality, surgical outcome, more often than it localized the SOZ to the same location (Hunyadi et al., 2015a). This Hunyadi et al. (2015a) study required that participants have an EEG-fMRI negative for localizing information, so this is a potential explanation for the lack of helpful information from a similarly BOLD-dependent modality (Hunyadi et al., 2015a). Though with it being such a relatively small study, caution to any conclusion is warranted. (5) Finally, somewhat spuriously different than the other studies, but in the opposite direction, Boerwinkle et al. (2019) had a relatively high odds ratio. A few key implementation aspects may yield some insight into this difference. One is that this study came from a repeat first author in relatively larger studies, implying possible influence of learned expertise effect. However, there is also the possibility of bias, though this is contradicted by a separate lab validating subjects from the same dataset using fully automated SOZ detection method with deep learning and artificial intelligence (Banerjee et al., 2023).

The random-effects analysis estimated a population median odds ratio of 1.007 (95% PI: 0.003–361.41). The hypothesis of homogeneity was strongly rejected (*p*-value = 0.001), *arguing against the existence of a true common odds ratio.* The prior meta-analysis estimated positive agreement in SOZ localization between rs-fMRI and the comparators (Chakraborty et al., 2020) using a random-effects model and found moderate, yet not significant (*p* = 0.162), heterogeneity in odd ratios. The added scope of rs-fMRI SOZ analysis methods, validation modalities, and wide range of patient demographics across studies may have contributed to the increased evidence against a common odds ratio. One source of such study-level heterogeneity is evidenced by the relatively wide array of rs-fMRI SOZ analysis methods, and even within the same investigator over time a difference potentially due to increasing experience. Notably, there could be an early emerging trend of convergence as the two most frequently used in recent larger studies were the data-driven ICA and hypothesis-driven SBC. This is not to say there is evidence of superiority between the analysis methods. Furthermore, we posit that each method has its strengths and, when used appropriately in isolation or combination, may improve SOZ localization and surgical outcomes. For example, to verify whether comparative modalities’ SOZ candidates contain the true SOZ, ICA may be an ideal initial search approach since it may hone in on a smaller cadre of region(s). Then, hypothesis-driven techniques, such as SBC or effective connectivity, may further narrow the SOZ candidates (Grande et al., 2020; Jayakar et al., 2016; Tavares et al., 2017; Chassoux et al., 2018; Mullin et al., 2016).

### 4.2 Modality evaluation

There were indicators of differences in SOZ localization agreement between rs-fMRI and the modality groups. (1) The fixed-effects analysis demonstrated high evidence of systematic differences in agreement between rs-fMRI across modalities with the level of agreement being higher for surgical outcome when compared to EEG (*p* = 0.002) and iEEG (*p* = 0.007). (2) In addition, the analysis presented high evidence of systematic differences in agreement between rs-fMRI and the comparator between studies sharing the same modality. These results suggest that while an overall positive agreement was determined by the fixed-effects analysis, the level of agreement varied across modalities, and even between studies using the same modality.

### 4.3 Sensitivity and specificity

The random-effects model estimated a population mean sensitivity of 0.91. These results indicate that there is a high probability of the rs-fMRI localizing the SOZ given that the comparative modality also localized the SOZ. In contrast, the estimated population mean specificity was 0.09 which indicates a low probability of rs-fMRI not localizing the SOZ given that the comparative modality also did not localize the SOZ. Similarly, the study-specific predicted sensitivities were always higher than the predicted specificities. Similar results were observed in the fixed-effects analysis.

### 4.4 Strengths and limitations

Strengths of this study include the wide inclusion of rs-fMRI SOZ analysis methods and extent of validation modality subtypes. In addition, this study is the first meta-analysis to investigate the sensitivity and specificity of rs-fMRI for seizure onset localization. However, there are several limitations to consider.

First, while the comparative localizing modalities are established as standards of care, this study evaluated concurrent validity, which measures the level of agreement between two or more instruments in assessing the same outcome as opposed to other measures of validity (Higgins and Straub, 2006). Second, nearly half of the studies included in our analysis had small sample sizes (*n* < 10), which contributes to low precision and non-estimable odds ratios, and may limit the reliability of our pooled sensitivity and specificity estimates. The predominance of small case studies also raises the possibility of publication bias, as studies with positive or significant results are more likely to be published, potentially skewing the overall findings. This publication bias could result in an overrepresentation of positive outcomes, which may artificially inflate sensitivity and specificity values for rs-fMRI’s accuracy in SOZ localization.

Third, heterogeneity across studies represents a significant limitation. The heterogeneity test for study-level odds ratios in both random- and fixed-effects analyses yielded statistically significant results, suggesting that the studies included in the meta-analysis exhibit heterogeneity and provide strong evidence that a study-level common odds ratio does not exist. This could be due to multiple factors including the inclusion of multiple comparative modalities, different rs-fMRI software, seizure etiology, and patient populations. Furthermore, our QUADAS-2 analysis indicated that 12 out of 25 studies had an uncertain or high risk of bias in at least one domain; these studies were retained in our comparisons as excluding them could have further limited our dataset. However, these high-risk studies may have introduced additional bias, impacting the robustness of our meta-analysis.

Furthermore, this analysis included only studies published up to 2019, which excludes recent advancements in rs-fMRI methodologies. Since 2019, newer techniques such as improved data acquisition, processing algorithms, and network-targeted approaches have emerged, potentially enhancing the clinical applicability of rs-fMRI for SOZ localization. Future meta-analyses incorporating these newer methodologies will be critical to validate and extend our findings as rs-fMRI evolves as a tool for SOZ localization. Additionally, with time the ILAE surgical outcome classification system has evolved to become the most broadly recognized system to compare surgical outcomes. Our study is limited by retrospective collection of surgical outcomes classified under the Engel approach, which may be improved in the future by transition to the ILAE approach.

While this is the largest meta-analysis to date evaluating the agreement of SOZ localization between rs-fMRI with other localizing modalities, the overall number of studies remains limited. Therefore, it is important to interpret these results as guidance for future validation studies of rs-fMRI.

### 4.5 Further directions

Studies are needed to validate the use of rs-fMRI in localizing SOZ with comparative modalities, with special consideration to modalities that have high accuracy such as iEEG and surgical outcomes. Evaluating the convergent validity of rs-fMRI against gold-standard modalities may allow clinicians to localize SOZ while minimizing the degree of invasive iEEG verification risks. In addition, rs-fMRI is an increasingly accessible imaging modality as more healthcare centers acquire three Tesla MRI machines and the images can be sent to experts for analysis, or possibly fully automated SOZ localization (Jayakar et al., 2016). This compares to methods such as iEEG, which requires on-site expertise for electrode placement. Rs-fMRI could benefit patients in resource-deprived communities by providing them with increased access to expertise. Unfortunately, studies evaluating the convergent validity of rs-fMRI with modalities such as iEEG are limited due to the invasive nature of gold-standard procedures, limited funding and resources such as insurance reimbursement (Young et al., 2023), access to the expertise of advanced modalities, and difficulties related to the recruitment of individuals willing to participate in these studies.

## 5 Conclusion

Accurate presurgical localization of SOZ is important as it is a determining factor in the success of treating epilepsy for patients undergoing surgical resection. Rs-fMRI is an emerging modality for localizing SOZ, but limited research exists evaluating its agreement with other established localizing modalities, especially when considering a wide variety of rs-fMRI analysis methods and comparative modalities. This meta-analysis considered 25 studies that compared agreement in SOZ localization between rs-fMRI and other localizing modalities, including a wider range of rs-fMRI analysis subtypes and verification modalities than previous meta-analysis studies. Using the odds ratio as a measure of within-study agreement, the data provide strong evidence for considerable variation in agreement between rs-fMRI and the other modalities across studies. Agreement with rs-fMRI was highest for surgical outcomes, followed by EEG and iEEG. In addition, there is significant variation in agreement between studies using the same modality. A high sensitivity and a low specificity of SOZ location for rs-fMRI when compared to the comparative were also found. While this is the largest meta-analysis evaluating the agreement of SOZ localization between rs-fMRI with other localizing modalities to date, the number of studies included in the analysis is small; therefore, it is important to use the results as guidance for future validation studies of rs-fMRI.

## Data Availability

The datasets presented in this study can be found in online repositories. The names of the repository/repositories and accession number(s) can be found in the article/supplementary material.

## Footnotes

^1^ http://www.covidence.org

^2^ https://github.com/BoerwinkleLab/SOZMetaAnalysis

## Glossary

ALFF: Amplitude of low-frequency fluctuation
aMRI: Anatomical MRI
BOLD: Blood-oxygen-level-dependent
CI: Confidence interval
DOR: Diagnostic odds ratio
DPARSF: Data Processing Assistant for Resting-State fMRI
DRE: Drug-resistant epilepsy
EEG: Scalp electroencephalography
F: Female
fALFF: Fractional ALFF
FN: False negative
FP: False positive
FSL: FMRIB Software Library
GLM: General linear model
GSR: Global signal regression
HRF: Hemodynamic response function
hypo: Hypothesis-driven method
ICA: Independent component analysis
ICC: Intrinsic connectivity contrast
IDL: Interactive data language
iEEG: Intracranial EEG
ILAE: International League Against Epilepsy
M: Male
MEG: Magnetoencephalography
MELODIC: Multivariate Exploratory Linear Optimized Decomposition into Independent Components
ML: MATLAB
MRI: High-resolution magnetic resonance imaging
NaN: Not a number
NR: Not reported
OCEBM: Oxford Centre for Evidence-Based Medicine Levels of Evidence Criteria
PCA: Principal component analysis
PET: Positron emission tomography
posLR: Positive likelihood ratio
PRISMA: Preferred Reporting Items for Systematic Reviews and Meta-Analyses
QUADAS-2: Quality Assessment of Diagnostic Studies
ReHo: Regional homogeneity
REST: Resting-State fMRI Data Analysis Toolkit
rs-fMRI: Resting-state functional magnetic resonance imaging
SBC: Seed-based correlation
SEEG: Stereo encephalography
SOZ: Seizure onset zone
SPECT: Ictal single-photon emission computed tomography
SPM: Statistical parametric mapping
TCA: Temporal clustering analysis
TN: True negative
TP: True positive
